# Early childhood caries prevalence and associated factors among preschoolers aged 3–5 years in Xiangyun, China: A cross-sectional study

**DOI:** 10.3389/fpubh.2022.959125

**Published:** 2022-08-16

**Authors:** Mingshan Liu, Qianqian Song, Xiaoqin Xu, Guangyun Lai

**Affiliations:** ^1^Department of Stomatology, People's Hospital of Xiangyun Affiliated to Dali University, Dali, China; ^2^Department of Pediatric Dentistry, Shanghai Key Laboratory of Stomatology, Shanghai Ninth People's Hospital, Shanghai Jiao Tong University School of Medicine, College of Stomatology, Shanghai Jiao Tong University, National Center for Stomatology, National Clinical Research Center for Oral Diseases, Shanghai, China

**Keywords:** early childhood caries, dental caries, prevalence, preschool children, epidemiological study, associated factors

## Abstract

**Purpose:**

This study aimed to investigate the early childhood caries (ECC) prevalence among preschoolers aged 3-5 years in Xiangyun of Yunnan, China and explore the factors associated with the prevalence.

**Materials and methods:**

A cross-sectional survey including 3-5-year-old children was conducted in Xiangyun County, China, between September and November 2020. According to the dental examination standard of the WHO 2013 criteria, the presence of ECC was recorded. Besides the dental examination for children, their parents completed questionnaires about caries-related factors, including demographic variables, family socioeconomic status, feeding, and oral health-related habits, parental dental knowledge, and the condition of dental service utilization. SPSS Statistics 25.0 (IBM, Chicago, IL, USA) was used for data analysis. Statistical significance was set at *p* < 0.05.

**Results:**

The ECC prevalence among a sample of 1,280 children aged 3–5 years consisting of 665 boys and 615 girls in this study, was 74.3%, and the mean decayed-missing-filled teeth (dmft) was 4.9 ± 5.0. There were no statistically significant differences in the ECC prevalence between the sexes and among different ethnic groups. Children with different dietary and oral hygiene habits showed no significantly different prevalence of ECC. Logistic regression analysis showed that the most significantly associated factors were older age, lower family income, and worse parental perception of children's oral health status.

**Conclusion:**

The ECC prevalence among 3–5-year-old preschool children in Xiangyun was higher than the average national ECC prevalence in China. This study implies that more attention should be given to children's caries prevention from early childhood; oral health education and promotion should be intensified to reduce the ECC prevalence and improve the oral health status of children in Xiangyun.

## Introduction

Dental caries is the most common non-communicable disease among children (1). Early childhood caries (ECC) is characterized by the presence of one or more decayed (non-cavitated or cavitated lesions), missing (due to caries), or filled tooth surfaces in any primary tooth in a child under the age of six (2). ECC can result in a high risk of pain or discomfort, abscesses, caries risk in permanent dentition, impact children's oral health-related quality of life, and bring an economic burden to society (1).

Despite being preventable, ECC still has a high prevalence among children around the globe. A recent systematic review reported a combined ECC prevalence of 48% worldwide based on various studies concerned with the ECC prevalence that used the WHO criteria (3). Additionally, data abstracted from 72 worldwide studies between 1998 and 2018 revealed that the mean caries prevalence for 1-year-olds was 17% and significantly increased to 36% in 2-year-olds (4). According to the 4th Chinese National Oral Health Survey, the mean caries prevalence in 5-year-old children has increased from 66 to 70.9% in the past decade, suggesting ECC in Chinese children is still a problem worthy of attention (5).

ECC is a complex multifactorial risk disease. The etiology of ECC includes cariogenic microorganisms and dietary and host determinants, which are influenced by multiple behavioral, sociological, and environmental factors, such as parental oral-health knowledge, family income, and dental utilization (6, 7). Effective ECC management requires caries risk-based prevention approaches and policies (8). Thus, monitoring the prevalence of ECC and exploring associated factors is essential for planning services and policies to control the disease and promote children's oral health.

Previously, some regions of China have reported the ECC prevalence and associated factors (9–16). However, because China is a vast country with a large population that is composed of 56 ethnic groups, 55 minorities, and the dominant Han group, different regions in China have significant dietary and cultural differences, which may influence children's oral health status. Moreover, along with the rapid economic development in China, inequalities in children's health have occurred (17). Until now, the epidemiological data on the ECC prevalence in children from remote and rural areas of Southwest China are rare. Therefore, this study aimed to investigate the ECC prevalence in 3–5-year old children in Xiangyun of Yunnan, a region located in Southwest China that has several minorities and eliminated poverty in 2018 (18) and explore factors related to the prevalence, including demographics, family socioeconomic status, feeding, and oral health-related habits, parental dental knowledge, and the condition of dental service utilization. We hope that this study can provide useful basic information for establishing public oral-health-related policies and interventions by the local government.

## Materials and methods

### Study design and sample

This cross-sectional study was conducted between September and November 2020 in Xiangcheng Town, Xiangyun County, China. Before the study initiation, the sample size was calculated using Power Analysis & Sample Size (PASS) software 16.0 with a 95% confidence interval, 5% standard error, 62.5% prevalence (the average caries prevalence of Chinese children aged 3-5 years in 2015) (5), and a 20% non-response rate. The minimum required sample size was 473. In coordination with the 2020 National Oral Health Comprehensive Intervention Program for children's teeth fluoridization in China, this study used a two-stage stratified cluster sampling method. Xiangcheng Town, Xiangyun County, was divided into four geographical regions (eastern, southern, western, and northern). According to the kindergartens' size, one or two kindergartens were selected from each region. All the children from each kindergarten were selected using the following inclusion and exclusion criteria.

The inclusion and exclusion criteria of the present study were applicable for both children who participated in the study and their legal guardians/parents. Children in a designated range of age (3–5years) had to attend the class on the survey day and could cooperate with the examiner. Children's parents/guardians were able to understand the study and be willing to sign the informed consent. The exclusion criteria were: the legal guardian's failure to understand this survey; the presence of systemic diseases or mental disorders in the children.

The Ethics Committee of the People's Hospital of Xiangyun approved the survey protocol (No. 2020069). Written informed consents were obtained from the participants' legal guardians/parents before the survey.

### Date collection

#### Clinical examination

The presence of ECC was determined using the WHO 2013 criteria (19). With working experience of more than 3 years, six dentists from the Department of Stomatology of the People's Hospital of Xiangyun received theoretical and clinical operation training before the survey. The test of intra-examiner and inter-examiner was conducted based on the methods recommended by WHO (19). Each dentist examined a group of volunteers (30 preschoolers) and re-examined each child on the second day. The mean Kappa values for both the intra-examiner and inter-examiner were over 0.85, which met the examination requirements (19).

On the scheduled day, children were examined in the kindergarten, sitting on chairs. The trained dentists examined children with a plane mouth mirror and a probe under artificial light. According to WHO guidelines, caries prevalence was recorded as decayed-missing-filled teeth (dmft) > 0 (19). No radiographs were taken.

#### Questionnaire survey

The questionnaire was in Chinese and modified based on the 4th Chinese National Oral Health Survey questions (5). These questionnaires were distributed and collected by teachers in each kindergarten who received unified training before the initiation of the field investigation. With the consent form, the parents or guardians were asked to complete the questionnaire the day before the clinical examination of their children. The questionnaire contained the following information:

Demographics (children's age, gender, single child, primary caregiver).
Family socioeconomic status (parental education level, family income).
Feeding and oral health-related habits and dental service utilization (feeding type within six months after birth, bedtime bottle before children aged 3, frequency of consuming desserts, sweet drinks and candies or chocolates, habit of eating snacks without toothbrushing before bed, children's age of starting brushing teeth, frequency of brushing teeth, parental supervision for brushing teeth, the use of fluoride toothpaste, dental floss, history of dental visit, and the application of fluoride varnish).
Parental oral health awareness and knowledge (parents' perception of children's oral health status; the knowledge regarding the importance of oral health, the treatment necessity of decayed primary teeth, the protections to teeth, and too much consumption of sweets leading to tooth decay).

### Data analysis

The presence or absence of ECC was the primary outcome variable. Categorical variables were expressed as numbers and percentages (%). Univariate analyses used the chi-square test to assess the differences between the ECC and caries-free groups. Variables showing significant associations were included in a logistic regression analysis model. All data were analyzed using SPSS Statistics 25.0 (IBM, Chicago, IL, USA). A *p*-value less than 0.05 indicated statistical significance. The average dmft score was exhibited in mean ± SD. The frequencies of dmft scores among different age groups were presented in a figure.

## Results

Among 1,764 children aged 3–5 years attending class to receive fluoride varnish for their teeth, 1,293 children consisting of 673 boys and 620 girls participated in the dental examination with consent from parents or guardians. And their parents completed the questionnaire. There were no withdrawals in the study. However, due to insufficient data in the questionnaire, such as family income, 13 children were excluded. Finally, data of 1,280 children, comprised of 665 (52.0%) boys and 615 (48.0%) girls aged 3–5 years, were analyzed (Table 1). The mean age of this sample was 4.3 ± 0.7 years.

**Table 1:** Prevalence of ECC and socioeconomic factors (*N* = 1280).

**Variables**	**N**	**%**	**Groups**		***P*-value**
			**Caries-free (N/%)**	**ECC (N/%)**	
**Sex**
Male	665	52.0%	176 (26.5%)	489 (73.5%)	0.516
Female	615	48.0%	153 (24.9%)	462 (75.1%)	
**Age (year)**
3	202	15.8%	71 (35.1%)	131 (64.9%)	<0.0001^*^
4	498	38.9%	142 (28.5%)	356 (71.5%)	
5	580	45.3%	116 (20.0%)	464 (80.0%)	
**Ethnicity**
Han	1,004	78.4%	262 (26.1%)	742 (73.9%)	0.54
Others	276	21.6%	67 (24.3%)	209 (75.7%)	
**Single child**
Yes	374	29.2%	99 (26.5%)	275 (73.5%)	0.686
No	906	70.8	230 (25.4%)	676 (74.6%)	
**Primary caregiver**
Parents	1,075	84.0%	278 (25.9%)	797 (74.1%)	0.768
Others	205	16.0%	51 (24.9%)	154 (75.1%)	
**Parental education level**
Middle school or below	457	35.7%	108 (23.6%)	349 (76.4%)	0.025^*^
High school	325	25.4%	75 (23.1%)	250 (76.9%)	
College	223	17.4%	56 (25.1%)	167 (74.9%)	
Undergraduate or above	275	21.5%	90 (32.7%)	185 (67.3%)	
**Family income (Yuan, per month)**
<6,000 Yuan	679	53.0%	159 (23.4%)	520 (76.6%)	0.006^*^
≧6,000 and <12,000	354	27.7%	87 (24.6%)	267 (75.4%)	
≧12,000	247	19.3%	83 (33.6%)	164 (66.4%)	
**Total**	1,280	100%	329 (25.7%)	951 (74.3%)	

ECC, early childhood caries.

* Statistically significant at P < 0.05.

**Figure 1:**
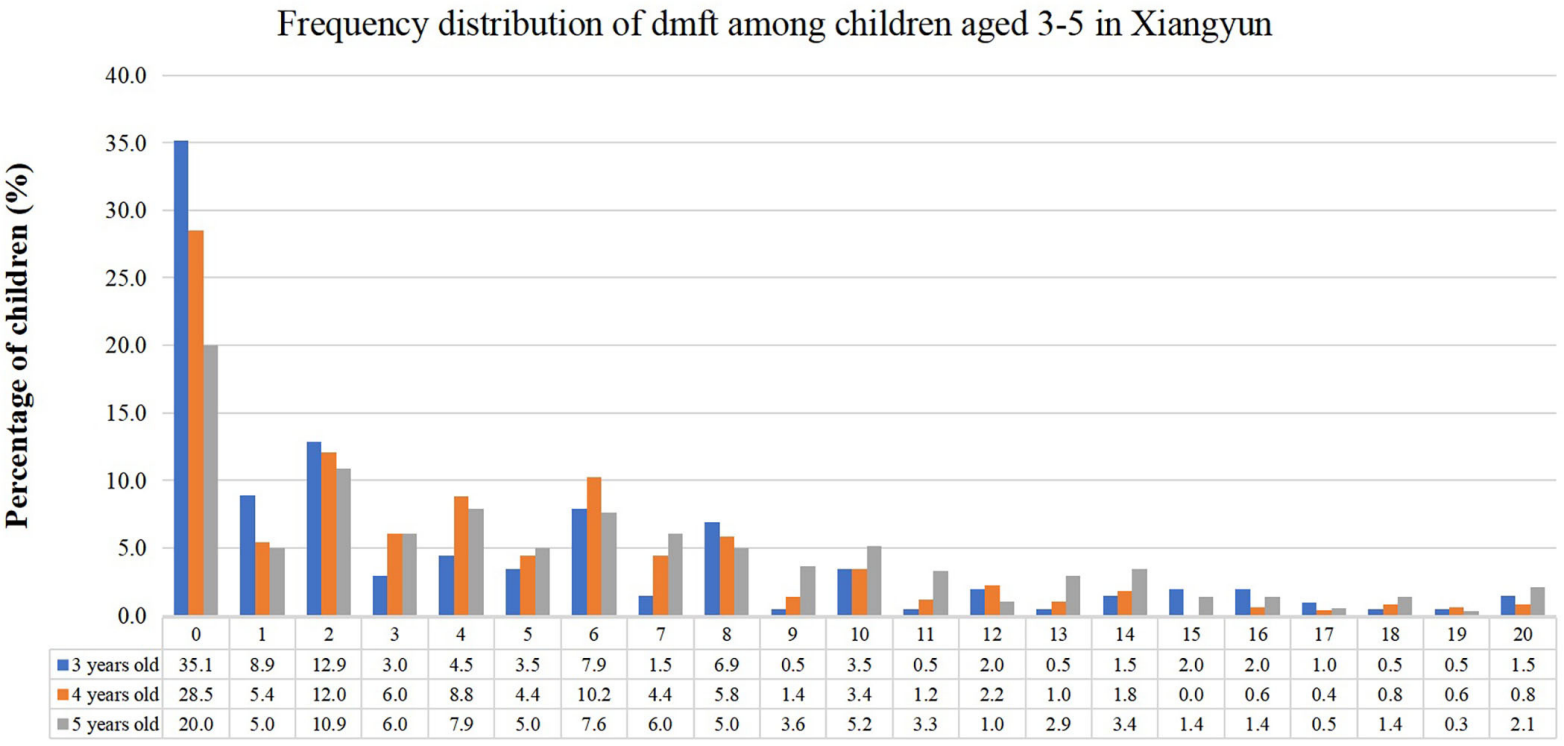
Frequency distribution of dmft among 3–5-year old children in Xiangyun, China.

The statistical results showed that the ECC prevalence of this sample was 74.3%, and the mean dmft was 4.9 ± 5.0. The mean dmft in each age group was 4.1 ± 5.1 in children aged three years, 4.3 ± 4.5 in children aged four years, and 5.7 ± 5.2 in children aged 5 years. The frequency distribution of dmft is displayed in Figure 1. Among all the children with ECC, children with two decayed teeth were the most common. In 3-year-old children with ECC, children with one decayed tooth were the second most common. On the contrary, in 4- and 5-year-old children with ECC, children with six decayed teeth were the second most common.

The ECC prevalence was 64.9% for 3-year-old children, 71.5% for 4-year-old children, and 80.0% for 5-year-old children, respectively. There were significant differences in the prevalence among age groups (*p* < 0.0001). Although the prevalence was slightly higher in girls (75.1%) than in boys (73.5%), the difference was statistically insignificant (*p* = 0.516). Han ethnic children showed a slightly lower prevalence than other ethnic children without significant differences (73.9 vs. 75.7%, *p* = 0.54). Similarly, single children showed no significant difference in ECC prevalence compared with non-single children (73.5 vs. 74.6%, *p* = 0.686).

Regarding the socioeconomic factors, children whose parents had an undergraduate degree or above showed significantly lower ECC prevalence (67.3%) than other children (76.4% for the parental education level of middle school degree or below, 76.9% for the parental education level of high school degree and 74.9% for the parental education level of college, respectively). The ECC prevalence in children from families with a monthly income of 12,000 Yuan and above was 66.4%, significantly lower than in other children (*p* = 0.006).

Table 2 shows that 561 (43.8%) children were breastfed within 6 months old and showed a slightly lower ECC prevalence (72%) than 719 (56.2%) children who had mixed-feeding or bottle-feeding without statistical significance (*p* = 0.099). Among all the children, 448 (35%) children experienced bedtime bottles before age 3; 425 (33.2%) children ate desserts at least once a day but showed a similar ECC prevalence (74.6%) to children who occasionally or never ate dessert (74.2%); 126 (9.8%) children consumed sweet drinks, and 326 (25.5%) ate candies/chocolates more than once a day. However, 897 (70.1%) children experienced consuming snacks before bed without toothbrushing. Regarding oral hygiene, 810 (63.3%) children started brushing their teeth at the age of 3 and even older; 652 (50.9%) children brushed their teeth twice a day, but they did not show lower ECC prevalence. Only 353 (27.6%) children used fluoride toothpaste, and 121 (9.5%) used dental floss. Additionally, 577 (45.1%) children had dental visit history and significantly higher ECC prevalence than the left 703 (54.9%) children (76.9 vs. 72.1%, *p* = 0.049).

**Table 2:** Prevalence of ECC and feeding history, dietary habit, oral hygiene behaviors and dental history (*N* = 1280).

**Variables**	**N**	**%**	**Groups**		***P*-value**
			**Caries-free (N/%)**	**ECC (N/%)**	
**Feeding type within 6-month after birth**
Breast only	561	43.8%	157 (28.0%)	404 (72.0%)	0.099
Other types	719	56.2%	172 (23.9%)	547 (76.1%)	
**Bedtime bottle before children aged 3**
Yes	448	35.0%	111 (24.8%)	337 (75.2%)	0.578
No	832	65.0%	218 (26.2%)	614 (73.8%)	
**Frequency of consuming desserts**
At least once a day	425	33.2%	108 (25.4%)	317 (74.6%)	0.866
Occasionally or never	855	66.8%	221 (25.8%)	634 (74.2%)	
**Frequency of consuming sweet drinks**
At least once a day	126	9.8%	32 (25.4%)	94 (74.6%)	0.934
Occasionally or never	1,154	90.2%	297 (25.7%)	857 (74.3%)	
**Frequency of consuming candies or chocolates**
At least once a day	326	25.5%	84 (25.8%)	242 (74.2%)	0.976
Occasionally or never	954	74.5%	245 (25.7%)	709 (74.3%)	
**Eating snacks without toothbrushing before bed**
Yes	897	70.1%	218 (24.3%)	679 (75.7%)	0.079
No	383	29.9%	111 (29.0%)	272 (71.0%)	
**Start brushing teeth**
1-year-old and below	24	1.9%	6 (25.0%)	18 (75.0%)	0.742
2-year-old	446	34.8%	109 (24.4%)	337 (75.6%)	
3-year-old and above	810	63.3%	214 (26.4%)	596 (73.6%)	
**Frequency of brushing teeth**
Twice per day or above	652	50.9%	165 (25.3%)	487 (74.7%)	0.904
Once a day	533	41.6%	138 (25.9%)	395 (74.1%)	
Occasionally or never	95	7.4%	26 (27.4%)	69 (72.6%)	
**Parental supervision for brushing teeth**
Every time	146	11.4%	38 (26.0%)	108 (74.0%)	0.857
Occasionally	947	74.0%	246 (26.0%)	701 (74.0%)	
Never	187	14.6%	45 (24.1%)	142 (75.9%)	
**Fluoride toothpaste**
Yes	353	27.6%	101 (28.6%)	252 (71.4%)	0.142
No	927	72.4%	228 (24.6%)	699 (75.4%)	
**Dental floss**
Yes	121	9.5%	33 (27.3%)	88 (72.7%)	0.678
No	1,159	90.5%	296 (25.5%)	863 (74.5%)	
**History of dental visit**
Yes	577	45.1%	133 (23.1%)	444 (76.9%)	0.049^*^
No	703	54.9%	196 (27.9%)	507(72.1%)	
**Fluoride varnish**
Yes	420	32.8%	104 (24.8%)	316 (75.2%)	0.59
No	860	67.2%	225 (26.2%)	635 (73.8%)	
**Total**	1,280	100%	329 (25.7%)	951 (74.3%)	

ECC, early childhood caries.

* Statistically significant at P < 0.05.

As shown in Table 3, 773 (60.4%) parents believed that their children's oral health status was good, and their children exhibited a significantly lower ECC prevalence than others (68.4 vs. 83.2%, *p* < 0.0001). Besides, 289 (22.6%) children's parents were unsure whether decayed primary teeth needed treatment or believed primary teeth did not need treatment.

**Table 3:** Prevalence of ECC and parental oral health awareness and attitude (*N* = 1,280).

**Variables**	**N**	**%**	**Groups**		***P*-value**
			**Caries-free (N/%)**	**ECC (N/%)**	
**Parents' perception of children's oral health status**
Good	773	60.4%	244 (31.6%)	529 (68.4%)	<0.0001^*^
Fair or poor	507	39.6%	85 (16.8%)	422 (83.2%)	
**Oral health is important to life**
Yes	1230	96.1%	316 (25.7%)	914 (74.3%)	0.961
No	50	3.9%	13 (26.0%)	37 (74.0%)	
**Decayed primary teeth do not require treatment**
Disagree	991	77.4%	259 (26.1%)	732 (73.9%)	0.512
Agree/ unknown	289	22.6%	70 (24.2%)	219 (75.8%)	
**Teeth are born healthy or unhealthy, no correlation with the protections**
Disagree	1,139	89.0%	294 (25.8%)	845 (74.2%)	0.8
Agree/ unknown	141	11.0%	35 (24.8%)	106 (75.2%)	
**Too much consumption of sweets can lead to tooth decay**
Known	1,233	96.3%	322 (26.1%)	911 (73.9%)	0.084
Unknown	47	3.7%	7 (14.9%)	40 (85.1%)	
**Total**	1,280	100%	329 (25.7%)	951 (74.3%)	

ECC, early childhood caries.

* Statistically significant at P < 0.05.

According to the logistic regression analysis (Table 4), the prevalence of ECC was significantly higher in children aged 5 years (*p* < 0.0001, OR 2.008; 95% CI: 1.390–2.902), those whose family income is lower than 12,000 Yuan *(p* = 0.025; OR 1.450; 95% CI: 1.047–2.007), and those whose parents think the child's oral health is poor **(***p* < 0.0001, OR 2.227; 95% CI: 1.680–2.951).

**Table 4:** Logistic regression analysis of factors associated with the ECC prevalence.

**Variables**		**B**	**SE**	**Wald χ^2^**	**P**	**OR**
Age groups				15.087	0.001^*^	
	3-year-old					
	4-year-old	0.307	0.182	2.842	0.092	1.360 (0.951–1.943)
	5-year-old	0.697	0.188	13.794	<0.0001^*^	2.008 (1.390–2.902)
Parental education level	Undergraduate or above					
	College or below	0.130	0.141	0.848	0.357	1.138 (0.864-1.500)
Family income	≥12,000 Yuan					
	<12,000 Yuan	0.371	0.166	5.016	0.025^*^	1.450 (1.047–2.007)
Parental perceptions of children's oral health status	Good					
	Fair or poor	0.801	0.144	31.059	<0.0001^*^	2.227 (1.680–2.951)
History of dental visit	No					
	Yes	0.128	0.136	0.889	0.346	1.137 (0.871–1.484)

ECC, early childhood caries; B, regression coefficient; SE, standard error; Wald χ^2^, a chi-square value; P, significant level; OR, odds ratios.

* Statistically significant at P < 0.05.

Based on the results shown in Table 5, although parental education level was not associated with children's feeding type or sweet food consumption, it was significantly associated with the frequency of brushing teeth and parental supervision for brushing teeth (*p* = 0.002 and *p* < 0.0001). Among children whose parents had an undergraduate degree or above, 158 (57.5%) children brushed their teeth twice daily. Moreover, children whose parents had higher education levels tended to use fluoride toothpaste (*p* < 0.0001) and receive fluoride varnish (*p* = 0.011) and less likely to eat sacks without toothbrushing before bed (*p* = 0.005). Concerning the parents' dental knowledge, parents with higher education levels tended to know the necessity of treating decayed primary teeth (*p* = 0.004) and the correlation between teeth health and protections (*p* = 0.001).

**Table 5:** Parental education level and different variables (*N* = 1,280).

**Variables**	**Parental education level**				***P-*value**
	**Middle school or below**	**High school**	**College**	**Undergraduate or above**	
**Feeding type within 6-month**					
Breast only	200 (43.8%)	142 (43.7%)	94 (42.2%)	125 (45.5%)	0.907
Other types	257 (56.2%)	183 (56.3%)	129 (57.8%)	150 (54.5%)	
**Bedtime bottle before children aged 3**					
Yes	170 (37.2%)	122 (37.5%)	73 (32.7%)	83 (30.2%)	0.158
No	287 (62.8%)	203 (62.5%)	150 (67.3%)	192 (69.8%)	
**Frequency of consuming desserts**					
At least once a day	151 (33.0%)	113 (34.8%)	61 (27.4%)	100 (36.4%)	0.169
Occasionally or never	306 (67.0%)	212 (65.2%)	162 (72.6%)	175 (63.6%)	
**Frequency of consuming sweet drinks**					
At least once a day	41 (9.0%)	37 (11.4%)	23 (10.3%)	25 (9.1%)	0.684
Occasionally or never	416 (91.0%)	288 (88.6%)	200 (89.7%)	250 (90.9%)	
**Frequency of consuming candies or chocolates**					
At least once a day	116 (25.4%)	91 (28.0%)	43 (19.3%)	76 (27.6%)	0.099
Occasionally or never	341 (74.6%)	234 (72.0%)	180 (80.7%)	199 (72.4%)	
**Eating snacks without toothbrushing before bed**					
Yes	346 (75.7%)	217 (66.8%)	157 (70.4%)	177 (64.4%)	0.005^*^
No	111 (24.3%)	108 (33.2%)	66 (29.6%)	98 (35.6%)	
**Start brushing teeth**					
1-year-old and below	8 (1.8%)	7 (2.2%)	3 (1.3%)	6 (2.2%)	0.791
2-year-old	147 (32.2%)	116 (35.7%)	81 (36.3%)	102 (37.1%)	
3-year-old and above	302 (66.1%)	202 (62.2%)	139 (62.3%)	167 (60.7%)	
**Frequency of brushing teeth**					
Twice per day or above	202 (44.2%)	167 (51.4%)	125 (56.1%)	158 (57.5%)	0.002^*^
Once a day	222 (48.6%)	126 (38.8%)	87 (39.0%)	98 (35.6%)	
Occasionally or never	33 (7.2%)	32 (9.8%)	11 (4.9%)	19 (6.9%)	
**Parental supervision for brushing teeth**					
Every time	42 (9.2%)	20 (6.2%)	31 (13.9%)	53 (19.3%)	<0.0001^*^
Occasionally	348 (76.1%)	245 (75.4%)	155 (69.5%)	199 (72.4%)	
Never	67 (14.7%)	60 (18.5%)	37 (16.6%)	23 (8.4%)	
**Fluoride toothpaste**					
Yes	115 (25.2%)	63 (19.4%)	76 (34.1%)	99 (36.0%)	<0.0001^*^
No	342 (74.8%)	262 (80.6%)	147 (65.9%)	176 (64.0.%)	
**Dental floss**					
Yes	43 (9.4%)	24 (7.4%)	24 (10.8%)	30 (10.9%)	0.431
No	414 (90.6%)	301 (92.6%)	199 (89.2%)	245 (89.1%)	
**History of dental visit**					
Yes	196 (42.9%)	147 (45.2%)	103 (46.2%)	131 (47.6%)	0.631
No	261 (57.1%)	178 (54.8%)	120 (53.8%)	144 (52.4%)	
**Fluoride varnish**					
Yes	128 (28.0%)	103 (31.7%)	82 (36.8%)	107 (38.9%)	0.011^*^
No	329 (72.0%)	222 (68.3%)	141 (63.2%)	168 (61.1%)	
**Oral health is important to life**					
Yes	434 (95.0%)	309 (95.1%)	217 (97.3%)	270 (98.2%)	0.089
No	23 (5.0%)	16 (4.9%)	6 (2.7%)	5 (1.8%)	
**Decayed primary teeth do not require treatment**					
Disagree	329 (72.0%)	257 (79.1%)	178 (79.8%)	227 (82.5%)	0.004^*^
Agree/unknown	128 (28.0%)	68 (20.9%)	45 (20.2%)	48 (17.5%)	
Teeth are born healthy or unhealthy, no correlation with the protections^*^					
Disagree	386 (84.5%)	294 (90.5%)	207 (92.8%)	252 (91.6%)	0.001^*^
Agree/ unknown	71 (15.5%)	31 (9.5%)	16 (7.2%)	23 (8.4%)	
**Too much consumption of sweets can lead to tooth decay**					
Known	439 (96.1%)	311 (95.7%)	218 (97.8%)	265 (96.4%)	0.625
Unknown	18 (3.9%)	14 (4.3%)	5 (2.2%)	10 (3.6%)	

* Statistically significant at P < 0.05.

As shown in Table 6, although children from higher-income families tended to brush their teeth twice a day (*p* = 0.006) and use fluoride toothpaste (*p* = 0.029), they were also likely to consume more desserts (*p* < 0.0001) and candies/chocolates (*p* = 0.006). While 42.1% of children whose family income was more than 12,000 Yuan per month consumed desserts at least once a day, the percentage was 27.8% among children whose family income was less than 6,000 Yuan per month.

**Table 6:** Family income and different variables (*N* = 1280).

**Variables**	**Family income (Yuan, per month)**			***P-*value**
	** <6,000**	**≥6,000 <12000**	**≥12,000**	
**Feeding type within 6-month**				
Breast only	306 (45.1%)	154 (43.5%)	101 (40.9%)	0.521
Other types	373 (54.9%)	200 (56.5%)	146 (59.1%)	
**Bedtime bottle before children aged 3**				
Yes	249 (36.7%)	119 (33.6%)	80 (32.4%)	0.392
No	430 (63.3%)	235 (66.4%)	167 (67.6%)	
**Frequency of consuming desserts**				
At least once a day	189 (27.8%)	132 (37.3%)	104 (42.1%)	<0.0001^*^
Occasionally or never	490 (72.2%)	222 (62.7%)	143 (57.9%)	
**Frequency of consuming sweet drinks**				
At least once a day	60 (8.8%)	35 (9.9%)	31 (12.6%)	0.245
Occasionally or never	619 (91.2%)	319 (90.1%)	216 (87.4%)	
**Frequency of consuming candies or chocolates**				
At least once a day	153 (22.5%)	92 (26.0%)	81 (32.8%)	0.006^*^
Occasionally or never	526 (77.5%)	262 (74.0%)	166 (67.2%)	
**Eating snacks without toothbrushing before bed**				
Yes	495 (72.9%)	238 (67.2%)	164 (66.4%)	0.062
No	184 (27.1%)	116 (32.8%)	83 (33.6%)	
**Start brushing teeth**				
1-year-old and below	11(1.6%)	6 (1.7%)	7 (2.8%)	0.13
2-year-old	226 (33.3%)	119 (33.6%)	101(40.9%)	
3-year-old and above	442 (65.1%)	229 (64.7%)	139 (56.3%)	
**Frequency of brushing teeth**				
Twice per day or above	314 (46.2%)	193 (54.5%)	145 (58.7%)	0.006^*^
Once a day	309 (45.5%)	135 (38.1%)	89 (36.0%)	
Occasionally or never	56 (8.2%)	26 (7.3%)	13 (5.3%)	
**Parental supervision for brushing teeth**				
Every time	54 (8.0%)	43 (12.1%)	49 (19.8%)	<0.0001^*^
Occasionally	507 (74.7%)	265 (74.9%)	175 (70.9%)	
Never	118 (17.4%)	46 (13.0%)	23 (9.3%)	
**Fluoride toothpaste**				
Yes	168 (24.7%)	103 (29.1%)	82 (33.2%)	0.029^*^
No	511 (75.3%)	251 (70.9%)	165 (66.8)	
**Dental floss**				
Yes	60 (8.8%)	35 (9.9%)	26 (10.5%)	0.701
No	619 (91.2%)	319 (90.1%)	221 (89.5%)	
**History of dental visit**				
Yes	287 (42.3%)	166 (46.9%)	124 (50.2%)	0.072
No	392 (57.7%)	188 (53.1%)	123 (49.8%)	
**Fluoride varnish**				
Yes	198 (29.2%)	125 (35.3)	97 (39.3%)	0.008^*^
No	481 (70.8%)	229 (64.7%)	150 (60.7%)	
**Oral health is important to life**				
Yes	648 (95.4%)	347 (98.0%)	235 (95.1%)	0.087
No	31 (4.6%)	7 (2.0%)	12 (4.9%)	
**Decayed primary teeth do not require treatment**				
Disagree	492 (72.5%)	291 (82.2%)	208 (84.2%)	<0.0001^*^
Agree/ unknown	187 (27.5%)	63 (17.8%)	39 (15.8%)	
**Teeth are born healthy or unhealthy, no correlation with the protections**				
Disagree	586 (86.3%)	325 (91.8%)	228 (92.3%)	0.005^*^
Agree/ unknown	93 (13.7%)	29 (8.2%)	19 (7.7%)	
**Too much consumption of sweets can lead to tooth decay**				
Known	649 (95.6%)	343 (96.9%)	241 (97.6%)	0.291
Unknown	30 (4.4%)	11 (3.1%)	6 (2.4%)	

* Statistically significant at P < 0.05.

## Discussion

This study is the first cross-sectional investigation on the ECC prevalence among 3–5-year-old preschoolers in Xiangyun, China, which can fill the knowledge gap about ECC prevalence in China. The present study revealed that the caries prevalence and the mean dmft of ECC among children aged 3-5 years in Xiangyun (74.3%, 4.9) were much higher than the national average score (62.5%, 3.35) reported by the fourth national epidemiological survey (5). Moreover, the ECC prevalence in this sample is not only higher than results in recent surveys of relatively developed regions, such as Guangdong (68.3%) (12), Shanghai (between 29.38% and 50.25% among 3–5-year old children) (15), and Zhejiang (70.4%) (14), but also developing regions, such as Weifang (between 46.4 and 63.1% among 3–5-year old children) (16) and Guizhou (63.1%) in China (13). These results indicate that dental caries is a severe and urgent problem among children in Xiangyun, China.

A significant association between ECC prevalence and age existed in the present study. The reason may be that caries is a continuous and cumulative process, increasing with age in any population independent of gender, urbanization, and social status (20). In addition, the severity of caries occurrence may increase with the longer exposure time of the dentition to the etiologic factors of caries without proper intervention.

Socioeconomic status relating to family income and parental education level is a widely documented risk indicator for ECC (4, 21, 22). In this study, the chi-square test and logistic regression analysis showed that lower family income was significantly associated with higher ECC prevalence, which was also demonstrated in children from Japan (23), America (24), Australia (25), Italy (26), and Mongolia (27). Although the logistic regression did not certify a significant relationship between lower parental education level and higher ECC prevalence, children tended to have higher prevalence when their parents had a lower education level in this study. Kato et al. (23) showed that higher caries prevalence was associated with lower levels of parental education among 3-year-old Japanese children. Cianetti (26) reported that a lower parental educational level was related to a higher presence of caries among an Italian population of children aged 4-14 years. However, no association between parental education and caries prevalence existed in a sample from Mongolia (27). The inconsistency of results among various studies may be due to the differences in study methodology, such as the study design, sample size, the time of data collection, and the methods for assessing ECC (21, 28).

Socioeconomic factors may influence children's caries status through children's oral health behavior and parents' oral health knowledge and attitude (10). In this study, both higher parental education level and family income were related to the notably higher frequency of brushing teeth and parental supervision of brushing teeth, a higher proportion of children using fluoride toothpaste and receiving fluoride varnish, and better knowledge about whether decayed primary teeth need treatment and teeth need protections (Tables 4, 5). In general, parents with higher socioeconomic status will have much more opportunities to access health information, preferentially attend public dental services, and receive oral health advice (13, 21), which, in turn, can contribute to caries prevention for children. Nevertheless, high socioeconomic status did not correlate with good dietary habits for caries prevention in the present study. Furthermore, children from higher-income families consumed desserts and candies considerably more frequently. This may be because high family income influences oral health knowledge and attitude, but oral health knowledge and attitude fail to affect dietary behavior, which was also observed in a previous study (10).

A systematic review concluded that children exposed to a long duration of breastfeeding up to age 12 months had a reduced risk of caries (29). In this study, children being exclusively breastfed during the first half-year of life showed a relatively lower ECC prevalence without significance. On the contrary, some Chinese researchers reported that children exclusively/predominantly breastfed during the first half-year of life had a higher risk of ECC (12). The controversial results may be because, besides the feeding type, the existence and duration of nocturnal feeding can also affect children's caries status, which was not investigated in this study. Thus, in future studies, the feeding type and habits, such as nocturnal feeding, should be included to explore the associated factors of ECC.

Regular toothbrushing with a fluoridated paste is generally considered a fundamental self-care behavior for preventing caries and maintaining oral health (30, 31). According to a systematic review, children brushing their teeth less frequently have an increased risk of developing new carious lesions than those brushing more frequently, which was more pronounced in primary than permanent dentition (32). Additionally, parents play an important role in their children's oral health (33). Matsuyama (34) reported that lack of parental supervision was associated with children's unhealthy oral health behaviors and dental caries. However, neither children with higher frequent toothbrushing, using fluoride toothpaste, nor parental supervision showed significantly lower ECC prevalence in this study, which may be attributed to two reasons. First, 63.3% of the children started brushing their teeth at the age of 3 or above when decayed teeth had already existed in the oral cavity. Second, in addition to the brushing frequency, the brushing duration, method, and brushing effect have a cumulative effect on caries prevention (35), which was not included in the present study. Based on the current results, we did not know the actual oral hygiene status among the children, which may affect the exploration of the association between toothbrushing and caries occurrence.

The American Academy of Pediatric Dentistry (AAPD) recommends that the initial dental visit be scheduled within 6 months of the first primary tooth eruption but no later than 12 months of age (36, 37). Werneck et al. (38) investigated a sample of Portuguese-speaking immigrants in Toronto and found that a higher proportion of children with ECC than non-ECC children had not visited a dentist. Conversely, the study on preschool children in Monastir, Tunisia, showed a statistically significant association between higher ECC prevalence and history of dental visits (39), which was also observed among children in Southern Italy (40), Saudi preschool children in Riyadh (41) and children aged 3–5 years from some regions of China (9, 12). In this study, despite the insignificant association evaluated by the logistic analysis, children with dental visit history tended to have a higher ECC prevalence. This might reflect that most children in many countries, including China, visit a dentist since they have already experienced a dental health problem, which is therapeutic rather than preventive (12). Therefore, visiting a dentist only when a problem is perceived rather than for preventive dental checkups could be one significant risk indicator for ECC (40).

According to the questionnaire survey, most parents had positive attitudes toward oral health care. They were aware of the importance of oral health, which, however, did not coordinate with the high prevalence of ECC. It is also worth noting that when parents thought their child's oral health was fair or poor, the ECC prevalence of their children was higher, indicating that parents might know their child's exact oral health status but did not make alterations. In future studies, we will explore the reasons for this phenomenon. Moreover, in view of this situation, increasing parental awareness of the seriousness of ECC treatment and its effect on oral health-related quality of life may help parents make behavioral alterations for improving children's oral health; preventive programs for ECC should involve children, as well as parents to lessen the disease burden.

In addition to the limitations mentioned above, this study does not allow the determination of the causal relationship between associated factors and the results due to the cross-sectional design. Moreover, as Xiangyun County has several towns, the study sample was drawn from the central town, Xiangcheng Town, which could lead to selection bias. Studies on the whole county's sample are needed in the future. Because we used the WHO criteria without radiographs for the examination, initial lesions have been neglected. Besides, potential response bias may exist as the data from parents were retrospective rather than prospective. Longitudinal studies in this field are necessary.

## Conclusion

In this study, the ECC prevalence among 3–5-year-old preschool children in Xiangyun was 74.3%. The mean dmft score was 4.9 ± 5.0. Children's age of 5-year-old, family income lower than 12,000 Yuan, and worse parental perception of children's oral health are critical factors related to the higher caries prevalence in this sample. This study implies that more attention should be given to children's caries prevention from early childhood; public awareness of ECC should be increased through community initiatives, and parents should help their children to develop good eating and oral hygiene habits; oral health education and promotion should be intensified to reduce the ECC prevalence and improve the oral health status of children in Xiangyun.

## Data availability statement

The raw data supporting the conclusions of this article will be made available by the authors, without undue reservation.

## Ethics statement

The studies involving human participants were reviewed and approved by the Ethics Committee of the People's Hospital of Xiangyun (No. 2020069). Written informed consent to participate in this study was provided by the participants' legal guardian/next of kin.

## Author contributions

ML and QS wrote the manuscript. XX collected the data. GL conceived the idea, analyzed the data, and revised the manuscript. All authors read and approved the final version of the manuscript prior to submission.

## Funding

This study received support from the Research Program of People's Hospital of Xiangyun Affiliated to Dali University (DX2020SF02).

## Conflict of interest

The authors declare that the research was conducted in the absence of any commercial or financial relationships that could be construed as a potential conflict of interest.

## Publisher's note

All claims expressed in this article are solely those of the authors and do not necessarily represent those of their affiliated organizations, or those of the publisher, the editors and the reviewers. Any product that may be evaluated in this article, or claim that may be made by its manufacturer, is not guaranteed or endorsed by the publisher.

## References

[1] Ending childhood dental caries: WHO implementation manual. Available online at: https://www.who.int/publications/i/item/ending-childhood-dental-caries-who-implementation-manual (accessed May 23, 2022).

[2] American Academy of Pediatric Dentistry. Policy on early childhood caries (ECC): classifications, consequences, and preventive strategies. Pediatr Dent. (2016) 38:52–4. 27931420

[3] Uribe SE Innes N Maldupa I. The global prevalence of early childhood caries: a systematic review with meta-analysis using the WHO diagnostic criteria. Int J Paediatr Dent. (2021) 31:817–30. 10.1111/ipd.12783 33735529

[4] Tinanoff N Baez RJ Diaz Guillory C Donly KJ Feldens CA McGrath C. Early childhood caries epidemiology, aetiology, risk assessment, societal burden, management, education, and policy: global perspective. Int J Paediatr Dent. (2019) 29:238–48. 10.1111/ipd.12484 31099128

[5] Wang X. Report of the Fourth National Oral Health Epidemiological Survey in China. Beijing: People's Medical Publishing House (2018). 228 p.

[6] Uribe S. Early childhood caries–risk factors. Evid Based Dent. (2009) 10:37–8. 10.1038/sj.ebd.6400642 19561571

[7] Anil S Anand PS. Early childhood caries: prevalence, risk factors, and prevention. Front Pediatr. (2017) 5:157. 10.3389/fped.2017.00157 28770188 PMC5514393

[8] Ng MW Chase I. Early childhood caries: risk-based disease prevention and management. Dent Clin North Am. (2013) 57:1–16. 10.1016/j.cden.2012.09.002 23174607

[9] Li Y Wulaerhan J Liu Y Abudureyimu A Zhao J. Prevalence of severe early childhood caries and associated socioeconomic and behavioral factors in Xinjiang, China: a cross-sectional study. BMC Oral Health. (2017) 17:144. 10.1186/s12903-017-0432-z 29197365 PMC5712104

[10] Qin Y Zhang R Yuan B Xu T Chen H Yang Y. Structural equation modelling for associated factors with dental caries among 3-5-year-old children: a cross-sectional study. BMC Oral Health. (2019) 19:102. 10.1186/s12903-019-0787-4 31170956 PMC6554934

[11] Zhang K Li J Lu Z. The prevalence of dental caries in primary dentition in 3- to 5-year-old preschool children in Northern China. Biomed Res Int. (2020) 2020:5315236. 10.1155/2020/5315236 32461999 PMC7238319

[12] Li J Fan W Zhou Y Wu L Liu W Huang S. The status and associated factors of early childhood caries among 3- to 5-year-old children in Guangdong, Southern China: a provincial cross-sectional survey. BMC Oral Health. (2020) 20:265. 10.1186/s12903-020-01253-w 32977784 PMC7517683

[13] Guan M Nada OA Wu JJ Sun JL Li N Chen LM. Dental caries and associated factors in 3-5-year-old children in Guizhou Province, China: an epidemiological survey (2015–2016). Front Public Health. (2021) 9:747371. 10.3389/fpubh.2021.747371 34660522 PMC8514823

[14] Zhou N Zhu H Chen Y Jiang W Lin X Tu Y. Dental caries and associated factors in 3 to 5-year-old children in Zhejiang Province, China: an epidemiological survey. BMC Oral Health. (2019) 19:9. 10.1186/s12903-018-0698-9 30630468 PMC6329098

[15] Su H Yang R Deng Q Qian W Yu J. Deciduous dental caries status and associated risk factors among preschool children in Xuhui District of Shanghai, China. BMC Oral Health. (2018) 18:111. 10.1186/s12903-018-0565-8 29921269 PMC6009057

[16] Jiang YY. Prevalence of early childhood caries among 2- to 5-year-old preschoolers in kindergartens of Weifang City, China: a cross-sectional study. Oral Health Prev Dent. (2017) 15:89–97. 28232979 10.3290/j.ohpd.a37718

[17] Quan JK Wang XZ Sun XY Yuan C Liu XN Wang X. Permanent teeth caries status of 12- to 15-year-olds in China: findings from the 4th National Oral Health Survey. Chin J Dent Res. (2018) 21:181–93. 30255169 10.3290/j.cjdr.a41080

[18] The goal of poverty elimination in Xiangyun was realized on schedule. Available online at: http://www.xiangyun.gov.cn/xyxrmzf/c102086/202011/b72579bd31b246c8aff44b2e607ce6db.shtml (accessed May 12, 2022).

[19] World Health Organization. Oral health surveys: basic methods. Geneva: World Health Organization (2013). 125 p.

[20] Chopra A Rao NC Gupta N Vashisth S Lakhanpal M. The predisposing factors between dental caries and deviations from normal weight. N Am J Med Sci. (2015) 7:151–9. 10.4103/1947-2714.156011 25973402 PMC4426518

[21] Zhang T Hong J Yu X Liu Q Li A Wu Z. Association between socioeconomic status and dental caries among Chinese preschool children: a cross-sectional national study. BMJ Open. (2021) 11:e042908. 10.1136/bmjopen-2020-042908 34020971 PMC8144044

[22] Schwendicke F Dörfer CE Schlattmann P Foster Page L Thomson WM Paris S. Socioeconomic inequality and caries: a systematic review and meta-analysis. J Dent Res. (2015) 94:10–8. 10.1177/0022034514557546 25394849

[23] Kato H Tanaka K Shimizu K Nagata C Furukawa S Arakawa M. Parental occupations, educational levels, and income and prevalence of dental caries in 3-year-old Japanese children. Environ Health Prev Med. (2017) 22:80. 10.1186/s12199-017-0688-6 29237397 PMC5729505

[24] Slade GD. Sanders AE. Two decades of persisting income-disparities in dental caries among US children and adolescents. J Public Health Dent. (2018) 78:187–91. 10.1111/jphd.12261 29243816 PMC6003830

[25] Jamieson LM Armfield JM Roberts-Thomson KF. Oral health inequalities among indigenous and nonindigenous children in the Northern Territory of Australia. Commun Dent Oral Epidemiol. (2006) 34:267–76. 10.1111/j.1600-0528.2006.00277.x 16856947

[26] Cianetti S Lombardo G Lupatelli E Rossi G Abraha I Pagano S. Dental caries, parents educational level, family income and dental service attendance among children in Italy. Eur J Paediatr Dent. (2017) 18:15–8. 28494596 10.23804/ejpd.2017.18.01.03

[27] Chinzorig T Aida J Cooray U Nyamdorj T Mashbaljir S Osaka K. Inequalities in caries experience among Mongolian children. Int J Environ Res Public Health. (2019) 16:E3892. 10.3390/ijerph16203892 31615100 PMC6843787

[28] Ndekero TS Carneiro LC Masumo RM. Prevalence of early childhood caries, risk factors and nutritional status among 3-5-year-old preschool children in Kisarawe, Tanzania. PLoS ONE. (2021) 16:e0247240. 10.1371/journal.pone.0247240 33630949 PMC7906390

[29] Tham R Bowatte G Dharmage SC Tan DJ Lau MX Dai X. Breastfeeding and the risk of dental caries: a systematic review and meta-analysis. Acta paediatrica. (2015) 104:62–84. 10.1111/apa.13118 26206663

[30] dos Santos AP Nadanovsky P de Oliveira BH. A systematic review and meta-analysis of the effects of fluoride toothpastes on the prevention of dental caries in the primary dentition of preschool children. Community Dent Oral Epidemiol. (2013) 41:1–12. 10.1111/j.1600-0528.2012.00708.x 22882502

[31] Raison H Corcoran R Harris RV. Is toothbrushing behaviour habitual? Cues, context, motivators and patient narratives. Commun Dent Oral Epidemiol. (2021) 49:478–86. 10.1111/cdoe.12624 33638565

[32] Kumar S Tadakamadla J Johnson NW. Effect of toothbrushing frequency on incidence and increment of dental caries: a systematic review and meta-analysis. J Dent Res. (2016) 95:1230–6. 10.1177/0022034516655315 27334438

[33] Bozorgmehr E Hajizamani A Malek Mohammadi T. Oral health behavior of parents as a predictor of oral health status of their children. ISRN Dent. (2013) 2013:741783. 10.1155/2013/741783 23738088 PMC3664493

[34] Matsuyama Y Isumi A Doi S Fujiwara T. Poor parenting behaviours and dental caries experience in 6- To 7-year-old children. Commun Dent Oral Epidemiol. (2020) 48:493–500. 10.1111/cdoe.12561 32750206 PMC7689935

[35] Alraqiq H Eddali A Boufis R. Prevalence of dental caries and associated factors among school-aged children in Tripoli, Libya: a cross-sectional study. BMC Oral Health. (2021) 21:224. 10.1186/s12903-021-01545-9 33931061 PMC8086357

[36] American Academy of Pediatric Dentistry. Perinatal and infant oral health care. Pediatr Dent. (2018) 40:216–20. 32074891

[37] Sanchez OM Childers NK. Anticipatory guidance in infant oral health: rationale and recommendations. Am Fam Physician. (2000) 61:115–20. 10643953

[38] Werneck RI Lawrence HP Kulkarni GV Locker D. Early childhood caries and access to dental care among children of Portuguese-speaking immigrants in the city of Toronto. J Can Dent Assoc. (2008) 74:805. 19000463

[39] Chouchene F Masmoudi F Baaziz A Maatouk F Ghedira H. Early Childhood Caries prevalence and associated risk factors in Monastir, Tunisia: a cross-sectional study. Front Public Health. (2022) 10:821128. 10.3389/fpubh.2022.821128 35284400 PMC8914024

[40] Nobile CG Fortunato L Bianco A Pileggi C Pavia M. Pattern and severity of early childhood caries in Southern Italy: a preschool-based cross-sectional study. BMC Public Health. (2014) 14:206. 10.1186/1471-2458-14-206 24571668 PMC3941481

[41] AlMarshad LK Wyne AH AlJobair AM. Early childhood caries prevalence and associated risk factors among Saudi preschool children in Riyadh. Saudi Dent J. (2021) 33:1084–90. 10.1016/j.sdentj.2021.04.003 34938053 PMC8665183

